# Impact of pregnancy on cancer survival: Experience at a tertiary care hospital

**DOI:** 10.12669/pjms.37.2.3525

**Published:** 2021

**Authors:** Dur-e-Shahwar, Sheikh Irfan, Zaheena Shamsul Islam, Lumaan Sheikh

**Affiliations:** 1 Dr. Dur-e-Shahwar FCPS. Department of Obstetrics and Gynaecology, Aga Khan University Hospital, Karachi, Pakistan; 2 Dr. Sheikh Irfan, Phd Fellow, MPH, MHP, MBBS. Department of Obstetrics and Gynaecology, Aga Khan University Hospital, Karachi, Pakistan; 3 Dr. Zaheena Shamsul Islam, FCPS. Department of Obstetrics and Gynaecology, Aga Khan University Hospital, Karachi, Pakistan; 4 Dr. Lumaan sheikh, FRCOG, FCPS. Department of Obstetrics and Gynaecology, Aga Khan University Hospital, Karachi, Pakistan

**Keywords:** Pregnancy, Cancer, Survival, Outcomes

## Abstract

**Objectives:**

To assess the overall survival of pregnant women diagnosed with cancer during pregnancy or became pregnant thereafter.

**Methods:**

A retrospective medical record review of 90 patients who were diagnosed with cancer when pregnant or who became pregnant thereafter between 1996 and 2015 in Aga Khan University Hospital, Karachi.

**Results:**

A total of 90 patients were analyzed. The malignancies that expectant mothers had were, breast cancer 38 (42.2%), hematological cancers 29 (32.2%), brain cancer 10 (11.1%), and other malignancies 13 (14.4%) that included thyroid cancers, gestational trophoblastic disease and synovial tumor of foot. We observed only four deaths out of 90 patients and mean survival time in pregnant patients with malignancies was 17.98 years [CI 16.35-19.31].

**Conclusions:**

The diagnosis of most cancer types before or during pregnancy does not influence on overall survival of patients.

## INTRODUCTION

Cancer is a leading cause of mortality worldwide, though a rare event during pregnancy. The annual incidence is reported to be approximately 1/1,000 pregnancies, which only makes 0.07% to 0.1% of all malignant tumors.^1^ This incidence is expected to increase due to delay in child bearing age. The common malignancies noted in pregnant women are brain, breast cancer, lymphomas, leukemia’s, cervical and ovarian cancer.^2^

The pathophysiology of cancer during pregnancy is poorly understood. Theoretically, hormonal and immunological variations with an increase in permeability and vascularization are thought to affect cancer prognosis and survival.^3^ Also, the outcomes are dependent on the type and stage of cancer. Few studies have reported a reduced survival rate in this group of women.^2^ However, there are contradictory findings in other studies.^4^,^5^

According to our knowledge no local data exists about survival in pregnant women with cancer. Hence the rationale of this study is to help our health care providers in the management of these women and enable them to counsel regarding prognosis and survival.

## METHODS

This was a retrospective observational study conducted in the department of Obstetrics & Gynecology, Aga Khan University Hospital Pakistan (AKUH). Medical records of the patients diagnosed with cancer during pregnancy and thereafter from 1996-2015 were reviewed in 2018. Inclusion criteria included all pregnant women with histologically confirmed malignant cancer before and during pregnancy with sufficient retrievable records code according to ICD-9 codes (International Classification of Diseases, 9th revision) system. The Hospital Information Management System (HIMS) at AKUH codes all inpatient medical records for diagnosis and procedure according (ICD-9-CM) coding system. Solid cancer Cases coded between 140.0 -195.8, whereas Hematological malignancies were coded from 2.200.00 to 209.29.

Data was collected on Pre-tested structured questionnaire after approval from Ethics Review Committee (ERC) of Aga Khan Hospital. Medical record files were reviewed for each case. Patient data comprised of basic demographic characteristics, obstetric/medical history, cancer diagnosis on registration, stages and grades of cancer, treatment administered and outcome. Data analysis was performed using SPSS version 19. Frequencies (percentages) were estimated for categorical variables. Overall survival analysis was conducted by using the Kaplan–Meier method.

### Operational Definition

Overall survival rate: Overall survival rate is percentage of people who survive a certain type of cancer for a specific period of time.^6^

## RESULTS

A total of 120 pregnant women with malignancies of different sites were registered at Aga Khan University Hospital between 1998 and 2015. Out of the total, 30 patient’s records had incomplete information regarding course of disease and therefore were excluded from the study. Five patients were lost to follow up. Ninety patients were included.

The common malignancies that expectant mothers had were; breast cancer 38(42.2%), hematological cancers 29(32.2%), brain tumor 10(11.1%), and other malignancies 13(14.4%) including thyroid cancers, gestational trophoblastic disease and synovial tumor of foot. (Table-I). We observed only 4 deaths out of 90 patients in our study. These deaths were found in patient with breast cancer and hematological malignancies.

**Table-I:** Characteristics of Patients (n=90).

	*Breast CA 38(42.2%)*	*Brain CA 10(11.1%)*	*Hem/Onc 29(32.2%)*	*Others 13(14.4%)*
***Age***				
<30 years	10 (25%)	5 (12.5%)	18 (45%)	7 (17.5%)
>30 years	28 (56%)	5 (10%)	11 (22%)	6 (12%)
***Parity***				
Primigravida	3 (30%)	1 (10%)	3 (30%)	3 (30%)
Multigravida (>2)	31 (49.2%)	6 (9.5%)	17 (27%)	9 (14.3%)
Grand multigravida (>5)	4 (23.5%)	3 (17.6%)	9 (52.9%)	1 (5.9%)
***Miscarriages***				
<2	36 (41.9%)	10(11.6%)	27(31.4%)	13(15.1%)
>2	2 (50%)	0	2 (50%)	0
***Treatment***				
Surgery	17 (60.7%)	6 (21.4%)	0	5 (17.9%)
Chemotherapy	21 (38.9%)	4 (7.4%)	23(42.6%)	6 (11.1%)
Hydroxyurea	0	0	6 (100%)	0
No treatment	0	0	0	2 (100%)
***Stage***				
Stage 1	10 (66.7%)	2 (13.3%)	0	3 (20%)
Stage 2	17 (58.6%)	5 (17.2%)	3 (10.3%)	4 (13.8%)
Stage 3	4 (44.4%)	2 (22.2%)	0	3 (33.3%)
Stage 4	7 (18.9%)	1 (2.7%)	26(70.3%)	3(8.1%)

Most of the patients were more than thirty years of age i.e. 50 (55.6%), however, majority women diagnosed with blood cancer patients were less than thirty years of age i.e., 18 (45%). More than half of the patients 63(70%) were multigravida. Approximately, 50% of women with breast and blood cancers also had history of more than two miscarriages. (Table-I).

Standard treatment modalities were used in all women with malignancies. 17 (60.7%) of patients with breast cancer, 6 (21.4%) of patients with brain tumor, 5 (17.9%) with other malignancies had surgery before pregnancy. While, 21 (38.9%) of breast cancer patients, 4 (7.4%) of patients with brain tumor, 23 (42.6%) of blood cancer patient, 6 (11.1%) of patients with other malignancies received chemotherapy.

**Fig.1:**
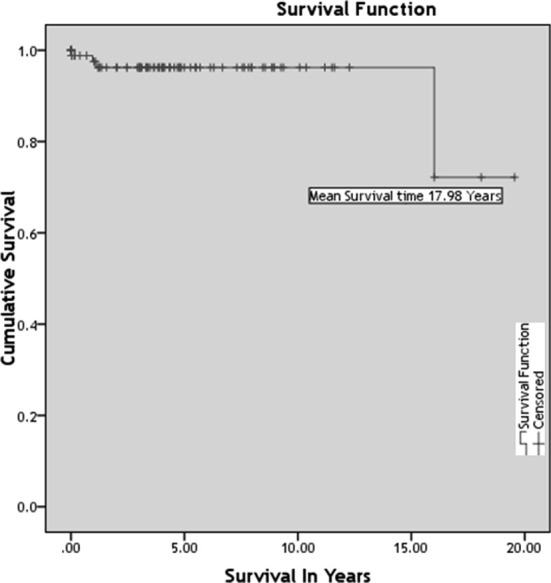
Survival analysis.

Most of pregnant patients with blood cancer were in staged IV i.e. 26 (70.3%). Most frequently found stage for breast cancer was stage I infiltrating ductal carcinoma i.e. 10 (66.7%). As shown in survival curve (Fig.1) mean survival time in the patients with malignancies was found to be 17.98 years [CI 16.35-19.31].

## DISCUSSION

The present study was performed to determine the common cancer types in pregnant women in our population and their characteristics and the effect of pregnancy on cancer survival in our setting. The cancer types which were commonly observed in our cohort were breast cancer, leukemias, lymphomas and brain tumors, consistent with the results of Palvidis et al.^7^

Till 2018 the cumulative survival time in our subjects was found to be 17.98 years. Which is consistent with one nationwide, registry-based study on most frequent cancer diagnoses during pregnancy and lactation. This confirms diagnosis of cancer during pregnancy or lactation does not increase the risk of cause-specific death compared with control group.

The most frequent tumor seen in these women was breast cancer. Majority of them were diagnosed during pregnancy. The reason might be as they have their first examination from a healthcare provider during pregnancy and thus are diagnosed late, which is contrary to the data from developed countries where national screening program are well established.^8^ In our patients with breast cancer, the most common type was Infiltrating Ductal Carcinoma (IDC), diagnosed at Stages I and II. Most of these patients received chemotherapy during second and third trimester whereas seventeen (60.7%) patients were planned to have breast cancer surgery at the time of cesarean section. Overall survival of this group showed healthy mother effect and these results are consistent with Iqbal J et al study that showed pregnancy did not adversely affect survival in women with breast cancer.^9^

In our study, hematological malignancies observed were leukemia, lymphomas and essential thrombocytosis. Due to some incomplete information, the effect of histological type and stage could not be assessed on overall survival rate of these patients. We had twenty nine women in this group, out of which nineteen cases were diagnosed before pregnancy and ten during pregnancy. Among these patients, twenty three (42.6%) received chemotherapy and six (100%) cases of essential thrombocytosis received hydroxyurea before pregnancy. From this group of patients, three women underwent termination of pregnancy as the chemotherapy was planned to start in the first trimester of pregnancy. Our findings suggest that prognosis of hematological malignancies during pregnancy is comparable to non-pregnant women, and usually good for those who received chemotherapy in the second half of pregnancy. This is in agreement with Hossam K et al findings^10^, That also embarked upon the importance of gestational age at diagnosis, stage of malignancy and teratogenicity of chemotherapeutic agent during pregnancy, if chemotherapy is necessary during first trimester then termination of pregnancy is generally indicated, But the outcome is usually good for those patients who receive chemotherapy in the second or third trimester.

The third most common cancer in our study was glial tumor of brain. It included, five patients with Grade-II astrocytoma, three had Grade-II and III oligodendroglioma and two had Grade-II ependymoma. Majority of them had post cancer pregnancies except two who were diagnosed during pregnancy with Grade-II astrocytoma and Grade-II oligodendroglioma, respectively. Out of total, six (21.4%) patients underwent surgery before pregnancy and four (7.4%) patients received chemotherapy. None of the patients had disease progression during pregnancy; which is consistent with Rønning PA study, that showed pregnancy have no impact on the survival of patients with Low grade gliomas.^11^

Other tumors included gestational trophoblastic disease, thyroid cancers and synovial foot tumor, all these pregnancies occurred after the treatment of the cancer. Similar to other studies regarding thyroid cancer and gestational trophoblastic diseases^12^,^13^ , we did not found statistically significant difference in overall survival for pregnant women.

There were four mortalities in our cohort. Two of these patients died of advanced stage breast cancer (stage IV and Stage III IDC, respectively). Both of these patients had full term deliveries with mean survival time of two years. The third mortality was of patient with Stage IV IDC who underwent termination of pregnancy at six weeks and survived for one year. This leads to belief that the extent of the disease at diagnosis has an impact on survival. Fourth case was in a woman with acute myeloid leukemia diagnosed in second trimester of pregnancy and delivered at term. She died after sixteen years due to sepsis.

The strength of our study is the compliance of our patients for long term follow up. The reason for this could be as the study was conducted in a private, fully-equipped, centrally-located, tertiary care hospital, also catering patients referred from remote areas.

### Limitations of the study

It includes limited number of patients and its retrospective nature. In a few cases the information was missing about certain details related to extent and course of the disease. However, it was not associated with significant impact on the overall survival analysis of these patients.

## CONCLUSIONS

The diagnosis of most cancer types before or during pregnancy has no influence on overall survival of patients.

### Authors Contribution:

**DS:** Designed, literature search, data collection, manuscript writing, editing of manuscript, responsible and accountable for the accuracy or integrity of the work.

**SI:** Did statistical analysis, manuscript writing and proof reading.

**ZSI:** Did review, editing and proof reading of manuscript.

**LS:** Conceptualization of topic, review and final approval of manuscript.
